# Soybean oil and probiotics improve meat quality, conjugated linoleic acid concentration, and nutritional quality indicators of goats

**DOI:** 10.1111/1750-3841.17669

**Published:** 2025-01-20

**Authors:** Yong Han, Defeng Wang, Wen Xiao, Chao Yuan, Yang Yang, Yong Long

**Affiliations:** ^1^ Guizhou University of Traditional Chinese Medicine Guiyang China; ^2^ Guizhou Institute of Animal Husbandry and Veterinary Science Guiyang China; ^3^ School of Animal Technology and Innovation, Institute of Agricultural Technology Suranaree University of Technology Nakhon Ratchasima Thailand

**Keywords:** biohydrogenation, fatty acid profile, meat quality, nutritional quality index, probiotics, soybean oil

## Abstract

This study aimed to investigate the impact of dietary soybean oil and probiotics on goat meat quality, total conjugated linoleic acids (TCLA) concentration, and nutritional quality indicators of goats. Thirty‐six male crossbred goats (Anglo‐Nubian♂× Thai native♀), weighing 18.3 ± 2.7 kg, were selected and randomly assigned to six groups in a 2 × 3 factorial design, with six replicates per group. The soybean oil supplementation levels were 25 and 50 g/kg, while the probiotic supplementation levels were 0, 2.5, and 5.0 g/h/day. The results showed that supplementing the diet with 50 g/kg soybean oil significantly improved the average daily gain (ADG) (*p* = 0.02) and carcass yield (*p* = 0.05), while reducing the feed conversion ratio (*p *= 0.05). Additionally, the addition of 2.5 g/h/day of probiotics significantly increased dry matter intake (*p*(*L*) = 0.05, *p*(*Q*) = 0.03). Notably, supplementation with 50 g/kg soybean oil reduced the Warner–Bratzler shear force (*p* = 0.05) and a* (*p* = 0.01) values of the *Longissimus thoracis et lumborum*. However, 2.5 g/h/day of probiotics significantly improved (*p*(*L*) = 0.01, *p*(*Q*) = 0.04) the a* value of *Longissimus thoracis et lumborum*. Soybean oil supplementation at 50 g/kg increased the ether extract composition of *Biceps brachii* (*p* = 0.05) and *Semimembranosus* (*p* = 0.05). Additionally, it significantly increased TCLA content (*p* < 0.01) and reduced the n−6/n−3 ratio (*p* < 0.01). Interestingly, the supplementation of 5.0 g/h/day probiotics significantly reduced the thrombogenic index (*p* = 0.03). Moreover, supplementing with 50 g/kg soybean oil (*p* = 0.03) and 5.0 g/h/day probiotics significantly improved the nutritive value index of goat muscle. Collectively, the findings suggest that the optimal supplementation levels of probiotics and soybean oil are 2.5 g/h/day and 50 g/kg, respectively. These levels have a more pronounced effect on improving the growth performance of growing goats, increasing CLA content, and enhancing meat quality.

## 1 INTRODUCTION

Goat meat is becoming increasingly popular due to its unique flavor, low cholesterol content, and high levels of conjugated linoleic acid (CLA), which has been reported to offer a wide range of health benefits, including anticancer, antiatherogenic, antidiabetic, and immunomodulatory function (Badawy et al., 2023; Ebrahimi et al., 2014). This has become a research hotspot for many feed formulators, who are looking for more effective methods to increase the production of these fatty acids (Cabiddu et al., 2017; Siurana & Calsamiglia, 2016). CLA is an intermediate produced during the biohydrogenation of alpha‐linolenic acid (ALA) and linoleic acid (LA) in the rumen (Thanh et al., 2021). During biohydrogenation, LA and ALA are naturally converted in the rumen to CLA, which is subsequently transformed into vaccenic acid (t‐11 C18:1, VA) and ultimately stearic acid as the final product (Bauman et al., 2020). The diverse potential physiological effects and health advantages of CLA primarily stem from the actions mediated by trans‐10, cis‐12‐CLA (t10, c12) and cis‐9, trans‐11‐CLA (c9, t11‐CLA) (Wu et al., 2023). Consequently, supplementing animal diets with vegetable or animal oils rich in LA and ALA has become a key research strategy for increasing the concentration of CLA in various meat products (Wang et al., 2020).

Numerous studies have demonstrated that supplementing ruminant diets with plant or animal oils rich in linolenic acid and LA, such as soybean, linseed, sunflower, and fish oils, can significantly increase the CLA content in beef and lamb products (Belon et al., 2021; Czauderna et al., 2012; Demirel et al., 2004; Mir et al., 2000; Shingfield et al., 2013; Verma et al., 2023; Wang et al., 2020). Husvéth et al. (2011) supplemented the diet of growing lambs with 5% soybean oil and sunflower oil, respectively. Their findings indicated that 5% soybean oil was more effective in increasing the levels of c‐9 and t‐11 CLA in *Biceps brachii*. Similarly, Oliveira et al. (2012) reported that supplementing the diet of Nellore young bulls with 3.8% soybean oil was more effective than 3.8% flaxseed oil in enhancing the CLA concentration in the *Longissimus thoracis et lumborum* of Nellore young bulls. However, studies by Beaulieu et al. (2002) and Fiorentini et al. (2018) noted that while supplementing cattle diets with soybean oil increased the intake of CLA in the rumen, it did not effectively enhance fatty acid deposition or the CLA content in meat.

Probiotics can indirectly enhance CLA levels in meat by influencing the abundance of related CLA‐producing microorganisms, making this an important strategy for increasing CLA content in meat (Özer & Kılıç, 2021; Wu et al., 2023). Among these microorganisms, *Lactobacillus acidophilus*, which constitutes 27% of the *Lactobacillus* genus, is the most common CLA‐producing strain. It converts LA into various hydroxy fatty acids that act as intermediates in CLA production (Wu et al., 2023). For example, *L. acidophilus* NBRC 13,951 has been shown to convert LA into 10‐hydroxy‐cis‐12‐octadecenoic acid (10‐HOE), a confirmed intermediate in CLA biosynthesis, through a multicomponent enzyme system (Ogawa et al., 2001; Oh et al., 2015). In addition, *Saccharomyces cerevisiae* primarily synthesizes the c9,t11‐CLA and t10,c12‐CLA isomers, though the production of these CLA isomers varies across strains (Wu et al., 2023). For instance, *Saccharomyces rouxii AKU 4118* and *American yeast AKU 4004* produce both t9,t11‐CLA and c9,t11‐CLA, whereas *Pichia farinose AKU 4262* and *Pichia wickerhamii AKU 4258* exclusively synthesized c9,t11‐CLA, with respective yields of 0.0154 and 0.0198 g/L, respectively (Ando et al., 2009). Cai et al. (2021) demonstrated that supplementing 0.6% *S. cerevisiae* in the diets of growing hybrid male goats improved rumen fermentation and growth performance. Furthermore, relevant studies have shown that *S. cerevisiae* and concentrate were mixed at a concentration ratio of 50 g/kg and then fed to Kamieniecka male lambs. Building on this, the supplementation of *S. cerevisiae* was carried out at an elevated ratio of 0.05 kg/h/day every 10 days in the subsequent trials. The results showed that *S. cerevisiae* significantly increased muscle quality parameter values and CLA levels in meat (Milewski & Zaleska, 2011). Additionally, Singh et al. (2016) found that supplementing the diets of Barbari lambs with 2.00 g/h/day of *S. cerevisiae* and *L. acidophilus* improved digestibility and growth performance, with *S. cerevisiae* demonstrating particularly strong effects.

To date, soybean oil and probiotics have been studied mainly in poultry and pig feed, with less research on ruminants. Additionally, studies on the impact of soybean oil and probiotics on meat fatty acid composition, particularly CLA concentration through microbial hydrogenation, are even scarcer. We hypothesized that goats fed a LA‐enriched diet and fed a probiotic diet containing *L. acidophilus* and *S. cerevisiae* would improve meat quality, fatty acid composition, and muscle CLA concentration. The objective was to investigate the effects of soybean oil and probiotics on goat meat quality, muscle fatty acid profile, and CLA concentration.

## 2 MATERIALS AND METHODS

### 2.1 Animals, materials, diets, and experimental design

The soybean oil and probiotics used were prepared once, and the fatty acid profile is presented in Table 1. The probiotics (trade name: Probiotics) were sourced from L.P. Feeds Tech (Thailand) Co., Ltd., and contained *L. acidophilus* (2.0 × 10¹^2^ cfu/g) and *S. cerevisiae* (5.0 × 10¹¹ cfu/g). The whole‐plant corn silage and concentrate were provided by the farm at Suranaree University of Technology.

**TABLE 1:** Fatty acid profile of the soybean oil that used in this study (g/100 g).

Items	Concentration
C14:0	0.37
C15:0	0.17
C16:0	9.14
C17:0	0.26
C18:0	3.89
C18:1	22.67
C18:2	50.35
C18:3	5.04
C20:0	1.69
C20:2	0.36
C22:0	1.67
C20:3n6	2.08
C23:0	0.22
C22:2	0.21
C20:5n3	1.28
C24:1	0.18

Thirty‐six growing male crossbred goats (Thai native ♀ x Anglo‐Nubian ♂) with an average body weight of 18.3 ± 2.7 kg and 8 months of age were used in this study. The goats were randomly assigned to one of six treatments according to a 2 × 3 factorial organized in a completely randomized design with six goats in each treatment. The levels of soybean oil (B) were 25 and 50 g/kg of concentrate, and the levels of probiotics (P) were 0, 2.5, and 5.0 g/h/day. All treatment groups in this experiment are shown in Table 2. To meet the nutritional requirements of the goats, the basal diet was formulated according to the NRC (2007) guidelines, with its composition outlined in Table 3.

**TABLE 2:** Experimental design.

Groups	Goats (*n*)	BW (kg)	Treatments
I	6	18.31 ± 2.04	Basic diet + 25 g/kg (B)
II	6	18.41 ± 1.72	Basic diet + 25 g/kg (B) + 2.5 g/h/d (P)
III	6	18.34 ± 1.90	Basic diet + 25 g/kg (B) + 5.0 g/h/d (P)
IV	6	18.82 ± 2.71	Basic diet + 50 g/kg (B)
V	6	18.22 ± 2.30	Basic diet + 50 g/kg (B) + 2.5 g/h/d (P)
VI	6	18.40 ± 2.01	Basic diet + 50 g/kg (B) + 5.0 g/h/d (P)

*Note*: B: soybean oil; P: probiotics.

Abbreviation: BW: body weight.

**TABLE 3:** The nutritional composition of concentrate and whole plant corn silage (g/kg, air‐dry basis).

Items	B_1_	B_2_
Whole plant corn silage	545	400
Rice straw	150	230
Cassava pulp	40	40
Soybean meal	42.5	53
Rice bran	45	105
Wheat bran	55	50
Rapeseed meal	35	12
Molasses	25	20
Soybean oil	25	50
1% composite premix ^a^	5	5
CaHPO₄	10	1
CaCO₃	5	5
NaCl	5	5
Urea	12.5	16
Chemical composition^b^		
DM	880	900
OM	767.7	807.2
CP	141.5	140.6
EE	38.6	62.5
NDF	481	460
ADF	331.9	303.3
P	5.2	5.6
Ca	10.9	10.5
ME, MJ/kg	11.8	11.8

Abbreviations: B_1_, 25 g/kg soybean oil supplementation group; B_2_, 50 g/kg soybean oil supplementation group; ADF, acid detergent fiber; CP, crude protein; DM, dry matter; EE, ether extracts; ME, metabolizable energy; NDF, neutral detergent fiber; OM, organic matter.

^a^ The premix provides the following per kg of ration: vitamin A, 380–2500 KIU; vitamin D3, 180–350 KIU; vitamin E, 900–4000 IU; iron, 8000–35,000 mg; zinc, 7000–15,000 mg; copper, 1700–2500 mg; iodine, 100–900 mg; manganese, 5500–15,000 mg; cobalt, 40–200 mg; selenium, 30–50 mg; calcium, 5–25%; and phosphorus 3–9%; moisture content ≤ 8%.

^b^ ME values are calculated, and the remaining values of chemical composition are actual measured values.

Before the experiment, all goats were thoroughly disinfected, dewormed, and vaccinated in accordance with the farm's management system. They were housed in individual 0.9 × 1.4 m pens (36 pens), and each equipped with three small tanks: one for whole plant corn silage, one for concentrate, and one for water. Throughout the experiment, the goats had unrestricted access to purified water. Before feeding, mix soybean oil and probiotics thoroughly with all feed ingredients according to the feed formula. Feed twice a day, at 9:00 a.m. and 05:00 p.m. Before the second feeding, it was ensured that approximately 10% of the previous feed remained in the trough.

### 2.2 Growth performance

During the experimental period, goats were weighed before feeding every 15 days, and the average daily gain (ADG) was calculated at the end of the study. Moreover, the weight of the feed provided and the weight of the remaining feed from the previous feeding were accurately measured each time to calculate the dry matter intake (DMI). Finally, we calculated the final feed conversion ratio (FCR) following the formula FCR = DMI / ADG, based on the obtained ADG and DMI values.

### 2.3 Chemical analysis

Feed samples were collected using the quartering method, dried at 65°C, and then crushed, sieved, and stored for analysis. The chemical composition of the feed was analyzed according to the methods of the Association of Official Analytical Chemists (AOAC, 2003), including dry matter (DM) (Method No. 930.01), crude protein (CP) (Method No. 954.01), calcium and phosphorus (Method No. 935.13), and ether extract (EE) (Method No. 920.39). The neutral detergent fiber (NDF) and ADF content of the feed was analyzed using the method of Van Soest et al. (1991), in which the NDF content was analyzed without thermostable α‐amylase, but with sodium sulfite, and expressed without residual Ash.

The chemical composition of muscle, including CP (Method No. 981.10) and EE (Method No. 935.38), was analyzed according to the methods outlined by AOAC (2005).

### 2.4 Slaughter performance

On the final day of the experiment, following the principles of animal euthanasia, all goats were stunned with electricity and then bled. Slaughter body weight, carcass weight (CW), body wall thickness (BWT), and area of *Longissimus thoracis et lumborum* (ALD) were recorded at slaughter. The criteria for evaluating the carcass followed the guidelines outlined by USDA (1992). The carcass was split along the central vertebrae, and the left side was separated between the 12th and 13th ribs for measurements. The ALD (between the 12th and 13th ribs) and BWT (5 cm from the carcass midline between the 12th and 13th ribs) were determined. The ALD was analyzed using a LI‐COR portable planimeter (LI‐3000A, Beijing Ecotek Company Limited) based on the method described by Yáñez et al. (2006). Muscle samples were collected from the *Semimembranosus*, *B. brachii*, and *Longissimus thoracis et lumborum*. These were taken from the forequarter, hindquarter, and loin (from the 12th rib counted backward to the 8th rib) of the left side of the carcass. All samples were wrapped in sterile tin foil, placed in ziplock bags, the air was removed, and frozen at −20°C for further analysis.

### 2.5 Meat quality and chemical composition

The redness (a*), lightness (L*), and yellowness (b*) values of meat were measured three times for each muscle sample using a colorimeter (model Minolta CR400, Minolta Inc.). The hue angle and chroma were calculated according to Priolo et al. (2002). Specifically, the hue angle was determined as tan^−1^ (b*/ a*), and the chroma was calculated as $\sqrt{a{*}^{2}+b{*}^{2}}$. The Warner–Bratzler shear force (WBSF) of cooked meat samples (cooked to a peak internal temperature of 71°C) was analyzed with a TAXT2 texture analyzer (Texture Technologies Corp) with a crosshead speed of 3.5 mm/s. Each muscle sample (1 × 1 × 2 cm) was cut as parallel to the muscle fiber direction as possible in three replicates. The colors of the meat and perirenal fat samples were analyzed using a MINLTA electronic chroma meter (LORPILOT TRADE CO., LTD).

### 2.6 Fatty acid profile

The fatty acids in soybean oil were analyzed according to the method of Q. R. Xue and Liang (2008). Moreover, *Semimembranosus*, *B. brachii*, and *Longissimus thoracis et lumborum* samples from each goat were pooled (1:1:1) by the goat for fatty acid and CLA analysis. The fatty acid profile of the meat samples was analyzed using an HP6890 gas chromatograph (GC) (Scientific Equipment Source). The pretreatment of meat samples for gas chromatography analysis was performed following the methods of Cordain et al. (2002) and Bondia‐Pons et al. (2007).

Fatty acid analysis was performed using a GC equipped with a flame ionization detector and a 100 × 0.25 mm fused silica capillary column (SP2560, Supelco Inc). The column temperature was initially set at 70°C for 4 min, then increased at a rate of 13°C/min to 175°C over 27 min. It was further increased at 4°C/min to 215°C and held for 31 min. Nitrogen was used as the carrier gas at a flow rate of 60 mL/min, and the oven temperature was maintained at 250°C. Both the flame ionization detector and injection port temperature were set at 280°C, and a 1 µL sample was injected using a 10‐µL injector.

### 2.7 Nutritional quality index

Nutritional quality parameters were calculated using the fatty acid profile results. These parameters were estimated by the following formula:

Atherogenicity index (AI)=(C12:0+4×C14:0+C16:0)/∑UFA (Ulbricht & Southgate, 1991)

Thrombogenic index (TI) =(C14:0+C16:0+C18:0)/[(0.5×ΣMUFA)+(0.5×∑n−6)+(3×∑n−3)+(∑n−3/∑n−6)] (Ulbricht & Southgate, 1991)

Nutritive value index (NVI)=(C18:0+C18:1)/C16:0 (Y. Chen et al., 2016).

Healthy fatty Index (HFI) =[(ΣMUFA×2)+(n−6×4)+(n−3×8)+(n−6/n−3)](ΣSFA×1)+(ΣMUFA×0.5)+(n−6×0.25)+(n−3×0.125)+(n−6/n−3)] (Dal Bosco et al., 2024)

Hypocholesterolemic/Hypercholesterolemic (HH) =ΣUFA/[C12:0+(4×C14:0)+C16:0] (S. Chen et al., 2004)

### 2.8 Data analysis

Initially, Excel 2021 was used to arrange and record all of the raw data that was gathered for this study. The Shapiro–Wilk test was performed to verify that the data followed a normal distribution. Subsequently, all data were analyzed using a 2 × 3 factorial arrangement with the General Linear Model procedure in SAS 9.4. Orthogonal contrast analysis and Duncan's new multiple range test were employed to compare treatment means. The statistical model used was as follows:

$$\begin{array}{ccc} Y_{\mathrm{ijk}} & = & \mu +B_{i}+P_{j}+B\times P_{\mathrm{ij}}+R_{k}+\varepsilon_{\mathrm{ijk}}\\ & & i=1\;\mathrm{to}\;2;\;j=1\;\mathrm{to}\;3;\;k=1\;\mathrm{to}\;6 \end{array}$$

where *Y_ijk_* = observation k in level *i* of soybean oil and level *j* of probiotics, *μ* = population mean, *B_i_* = effect of level *i* of soybean oil, *P_j_* = effect of level *j* of probiotics, *B* × *P_ij_* = effect of the interactions of level *i* of soybean oil & level *j* of probiotics, *R_k_* = goat effect, and *ε_ijk_* = residual error. The means and standard error (SEM) of the means were used to express each result. In this study, *p* < 0.05 was regarded as statistically significant.

## 3 RESULTS AND DISCUSSION

### 3.1 Growth performance

After probiotics were supplemented in the diet; the DMI of growing goats exhibited a quadratic growth trend (*p*(*Q*) = 0.03). When probiotic supplementation exceeded 2.5 g/h/day, DMI decreased. Moreover, supplementation with 50 g/kg soybean oil significantly increased (*p* = 0.02) the ADG of goats. Notably, the FCR value of the diet supplemented with 50 g/kg soybean oil was significantly lower (*p* = 0.05) than that of the diet supplemented with 25 g/kg soybean oil (Table 4). Compared to the group supplemented with 25 g/kg soybean oil, 50 g/kg soybean oil significantly increased the ADG of goats, which could be attributed by the high DMI in the diet (Miltko et al., 2019). Previous studies have demonstrated that rumen microorganisms can adapt to diets regardless of the amount or composition of added edible oil. Additionally, the supply of roughage can mitigate the negative effects of low ruminal pH caused by excessive edible oil supplementation on rumen microorganisms (Messana et al., 2013; Ørskov, 1994). While edible oil supplementation typically reduces DMI, it generally does not affect ADG, which could explain the reduced FCR observed in this study. However, a higher ADG was recorded in the 50 g/kg soybean oil group compared to the 25 g/kg group, which requires further research and verification.

**TABLE 4:** Effects of soybean oil and probiotics supplementation on growth performance in goats.

Items	B_1_			B_2_			SEM	*p*‐value			
	P_1_	P_2_	P_3_	P_1_	P_2_	P_3_		B	P(L)	P(Q)	B × P
DMI (g/d)	416.31**^bc^**	507.90**^a^**	478.71**^ab^**	409.62**^c^**	500.82**^a^**	459.43**^b^**	39.30	0.63	0.05	0.03	0.97
ADG (g/d)	51.61**^B^**	54.44**^B^**	63.15	62.55**^A^**	65.86**^A^**	63.35	1.31	0.02	0.28	0.18	0.26
FCR	8.17**^A^**	8.55**^A^**	7.26	6.57**^B^**	7.62**^B^**	7.43	0.60	0.05	0.10	0.49	0.13

*Note*: B_1_ and B_2_ represent the soybean oil supplementation ratios of 25 g/kg and 50 g/kg, respectively; P_1_, P_2_, and P_3_ represent probiotic supplementation rates of 0, 2.5, and 5.0 g/h/day, respectively. P(L), linear of probiotics; P(Q), quadratic effect of probiotics; B × P, *p* value of cross‐interaction between soybean oil and probiotics. *p* < 0.05, significant. Different superscript letters within the same row denote significant differences, where lowercase letters (a, b, c…) represent probiotic supplementation levels and uppercase letters (A, B…) represent soybean oil supplementation levels. The rest of the tabular data descriptions in this study are presented in the same manner.

Abbreviations: ADG, average daily gain; DMI, dry matter intake; FCR, feed conversion ratio; SEM, standard error of the mean.

*S. cerevisiae* can change rumen fermentation patterns. Supplementing the diet with *S. cerevisiae* can eliminate the amount of dissolved oxygen in the rumen and enhance lactic acid metabolism, providing an optimal growth environment for cellulolytic bacteria, including *Ruminococcus* and *Fibrobacter succinogenes*. It also provides vitamins, such as biotin and thiamine, which support fungal activity and growth (Akin & Borneman, 1990; AlZahal et al., 2014; Zhu et al., 2017). Moreover, *S. cerevisiae* can also reduce the abundance of lactic acid‐producing bacteria while promoting the growth and reproduction of lactic acid‐utilizing bacteria, thereby regulating rumen pH, increasing DMI, facilitating the synthesis of microbial protein, and increasing VFA (mainly acetic acid, propionic acid, and butyric acid) concentration and N conversion rate (Chaucheyras‐Durand et al., 2008; Xiao et al., 2016; Zhu et al., 2017). Cai et al. (2021) and L. Xue et al. (2022) showed that supplementing the diet with 0.6% *S. cerevisiae* could improve the damage of heat stress to Boer crossbred goats and improve their growth performance. Additionally, it was concluded by Zhang et al. (2023) that supplementing the diet of dairy goats with 3.0 g/goat per day and 4.5 g/goat per day of *S. cerevisiae* could increase DMI by improving rumen cellulolytic enzyme activities, cellulolytic bacteria abundance, and nitrogen utilization. Finally, the ADG of goats was improved.

Similarly, *L. acidophilus*, as one of the important members of the *Lactobacillus* genus, serves as an important growth promoter for ruminant. It not only promotes the growth of lactic acid‐utilizing bacteria and alters the rumen fermentation pattern but also increases the abundance of cellulose‐decomposing bacteria, such as *Ruminococcus* (Cherdthong et al., 2021; Lettat et al., 2012). Additionally, it also has the ability to compete for nutrients and produce antimicrobial compounds in the gastrointestinal tract, occupies a specific spatial position in the gastric mucosa, and stimulates the activation of the innate immune system in young ruminants (Frizzo et al., 2011). Notably, related studies have shown that supplementing *L. acidophilus* in the diet can also reduce the amount of *Escherichia coli* in feces (Osborn, 2004). Mao et al. (2023) demonstrated that supplementation with probiotics containing *L. acidophilus* can improve growth performance, rumen fermentation, and microbial composition in growth‐retarded male Hu lambs. Furthermore, it was concluded by Jinturkar et al. (2009) that adding the diet of growing goats with 2 g/kg of *L. acidophilus* and 1 g/kg of *S. cerevisiae* significantly increased the ADG of goats.

In conclusion, this study found that the group supplemented with 50 g/kg soybean oil achieved a higher ADG compared to group B_1_. We speculate that this improvement may be partially attributed to the supplementation of probiotics. While the data did not show a significant direct effect of probiotics on ADG, their influence is likely indirect. Probiotics altered rumen fermentation patterns and microbial composition, leading to an increase in the DMI of goats. Additionally, soybean oil supplementation increased the proportion of propionate in the volatile fatty acids, a critical factor for goat growth. Ultimately, the ADG of goats was increased; still more in‐depth research in the future is needed to prove this speculation, particularly to gain a deeper understanding of the interaction between soybean oil and probiotics.

### 3.2 Slaughter performance

Soybean oil supplementation significantly increases (*p* = 0.05) carcass yield in goats. Conversely, gut rate significantly decreased (*p* = 0.03) by supplementation of soybean oil. Furthermore, after probiotics supplementation, the gut rate and ALD increased in a quadratic curve (*p*(*Q*) = 0.05) and then decreased when the supplementation rate exceeded 2.5 g/h/day (Table 5). We hypothesize that the improvement in carcass yield observed with soybean oil supplementation is primarily due to the enhancement of ADG. Our findings align with those reported by Awawdeh et al. (2009). Briefly, compared with the 25 g/kg soybean oil group, supplementing the diet with 50 g/kg soybean oil increased the final body weight of the animals by enhancing their ADG, ultimately improving slaughter performance. However, the reasons behind the reduced gut rate observed in this study require further investigation and verification.

**TABLE 5:** Effects of soybean oil and probiotics supplementation on slaughter performance in goats.

Items	B_1_			B_2_			SEM	*p*‐value			
	P_1_	P_2_	P_3_	P_1_	P_2_	P_3_		B	P(L)	P(Q)	B × P
SBW (kg)	20.91	20.64	21.75	20.17	21.35	21.44	2.06	0.93	0.77	0.81	0.87
CW (kg)	9.44	8.52	9.68	9.27	9.64	9.76	0.93	0.71	0.83	0.67	0.71
Leg score	12.04	12.02	11.53	12.04	12.05	12. 58	0.95	0.53	0.97	0.84	0.61
BWT (cm)	0.81	0.82	0.71	0.93	0.91	0.82	0.12	0.10	0.48	0.78	0.57
Carcass yield (%)	45.22**^B^**	43.02**^B^**	44.14**^B^**	47.43**^A^**	47.20**^A^**	46.31**^A^**	1.05	0.05	0.77	0.66	0.81
Fat rate (%)	1.32	1.55	1.97	1.91	2.15	2.03	0.45	0.10	0.61	0.82	0.70
Gut rate (%)	22.44**^Aab^**	23.88**^Aa^**	22.22**^Aab^**	18.94**^Bbc^**	19.12**^Bb^**	17.93**^Bc^**	2.72	0.03	0.67	0.05	0.98
ALD (cm^2^)	11.34**^ab^**	13.32**^a^**	10.83**^b^**	11.64**^ab^**	12.15**^ab^**	11.97**^ab^**	1.61	0.95	0.43	0.05	0.61

*Note*: Different superscript letters within the same row denote significant differences, where lowercase letters (a, b, c…) represent probiotic supplementation levels and uppercase letters (A, B…) represent soybean oil supplementation levels.

Fat rate = (kidney fat + pelvic fat + heart fat) / CW × 100.

Abbreviations: ALD, area of *Longissimus thoracis et lumborum*; BWT, body wall thickness; CW, carcass weight; SBW, slaughter body weight; SEM, standard error of the mean.

### 3.3 Meat quality

The a* value of the *Longissimus thoracis et lumborum* was significantly reduced (*p* = 0.04) after supplementing the diet with 50 g/kg soybean oil. However, the a* value of the *Longissimus thoracis et lumborum* was improved (*p*(*L*) = 0.01, *p*(*Q*) = 0.04) after probiotic supplementation. Additionally, supplementation with 50 g/kg soybean oil significantly reduced the a* value (*p* = 0.02) and b* value (*p* = 0.01) of perirenal fat compared to the B_1_ group. Interestingly, supplementation with 50 g/kg soybean oil (*p* = 0.04) and probiotics (*p* = 0.03) significantly reduced the chroma value of perirenal fat. Furthermore, in the *Longissimus thoracis et lumborum*, the hue angle value reached its lowest (*p* = 0.02) point at a probiotic supplementation level of 2.5 g/h/day, while the chroma value was highest (*p* = 0.04) at the same level (Table 6).

**TABLE 6:** Effects of supplementation with different soybean oils and probiotics on muscle and perirenal fat color in goats.

Items	B_1_			B_2_			SEM	*p*‐value			
	P_1_	P_2_	P_3_	P_1_	P_2_	P_3_		B	P(L)	P(Q)	B × P
*Biceps brachii*											
L*	41.01	42.61	48.03	42.54	43.68	42.92	5.19	0.77	0.61	0.89	0.61
a*	16.14	15.12	14.44	15.22	15.14	14.56	1.82	0.82	0.66	0.72	0.92
b*	8.34	8.02	7.24	7.92	8.24	7.12	1.18	0.94	0.45	0.60	0.92
Chroma	18.18	17.12	16.16	17.16	17.26	16.25	0.87	0.66	0.41	0.81	0.84
Hue angle	27.21	28.01	26.57	27.25	29.29	26.17	1.95	0.82	0.32	0.75	0.88
*Semimembranosus*											
L*	40.31	42.22	46.60	40.54	41.56	39.87	2.07	0.10	0.38	0.55	0.14
a*	15.51	16.92	14.35	14.74	15.18	13.66	1.02	0.12	0.11	0.09	0.77
b*	7.54	8.26	7.68	7.97	8.85	7.34	1.29	0.82	0.52	0.10	0.86
Chroma	17.26	18.85	16.26	16.77	17.57	15.50	0.91	0.42	0.56	0.61	0.62
Hue angle	25.80	25.78	28.33	28.49	30.56	28.32	2.21	0.12	0.24	0.58	0.71
*Longissimus thoracis et lumborum*											
L*	41.84	41.91	45.22	38.84	39.35	36.86	5.474	0.15	0.93	0.46	0.28
a*	14.97**^ab^**	18.73**^Aa^**	14.15**^Ab^**	14.21**^b^**	15.13**^aBb^**	12.74**^Bb^**	1.50	0.04	0.01	0.04	0.39
b*	8.10	8.11	8.12	8.30	7.44	9.32	1.14	0.75	0.53	0.28	0.51
Chroma	17.00**^ab^**	20.41**^a^**	16.28**^ab^**	16.46**^ab^**	16.87**^ab^**	15.79**^b^**	0.82	0.08	0.04	0.12	0.55
Hue angle	28.47**^bc^**	23.40**^c^**	30.52**^b^**	30.78**^b^**	26.27**^bc^**	36.47**^a^**	1.45	0.06	0.02	0.28	0.47
Perirenal fat											
L*	76.53	70.21	77.51	75.50	73.40	76.58	1.25	0.85	0.14	0.19	0.66
a*	7.11**^A^**	8.78**^A^**	7.37**^A^**	5.51**^B^**	5.43**^B^**	4.21**^B^**	1.75	0.02	0.58	0.33	0.73
b*	7.71**^Aab^**	9.25**^Aa^**	7.04**^Abc^**	6.06**^Bbc^**	5.74**^Bbc^**	4.70**^Bc^**	1.04	0.01	0.40	0.48	0.73
Chroma	10.49**^Aab^**	12.75**^Aa^**	10.19**^Aab^**	8.20**^Bbc^**	7.91**^Bbc^**	6.31**^Bc^**	0.68	0.04	0.03	0.52	0.65
Hue angle	47.47	46.70	43.52	47.72	46.61	48.72	2.15	0.19	0.06	0.42	0.55

*Note*: Different superscript letters within the same row denote significant differences, where lowercase letters (a, b, c…) represent probiotic supplementation levels and uppercase letters (A, B…) represent soybean oil supplementation levels.

Abbreviations: a*, redness; b*, yellowness; L*, lightness; SEM, standard error of the mean; WBSF, Warner–Bratzler shear force.

Color is a key attribute of meat appearance and an important factor influencing consumer purchasing decisions. Any color instability or change in fresh meat, which is affected by its pH, muscle fat content, and myoglobin content, will directly affect consumers' purchasing decisions. Generally, better meat quality is associated with higher a* values, whereas higher L* values indicate a paler color and lower meat quality (Lopes et al., 2014). On the other hand, hue angle can be expressed as the trueness of red, and high values are more intense brown. Moreover, the chroma value represents color saturation, with higher values reflecting more vivid colors and greater redness (Salim et al., 2022). Goat meat has lower L* and higher a* values compared to other meats, due to the lower intramuscular fat (IMF) content in their carcasses (Warner et al., 2010). However, probiotics indirectly promote fat deposition by altering the composition of rumen microorganisms, such as enhancing the growth of fiber‐degrading and lactic acid‐utilizing bacteria, thereby increasing DMI, nutrient digestibility, and VFA concentration, and promoting the synthesis of microbial protein (Cherdthong et al., 2021; Lettat et al., 2012; Xiao et al., 2016; Zhu et al., 2017). On the other hand, dietary supplementation with soybean oil directly promotes IMF deposition in muscle (Awawdeh et al., 2009; Saqhir et al., 2012). To our knowledge, this will lead to more nutrients being converted into muscle or fat reserves, which means more energy and nutrients are available for the synthesis of IMF. In this study, supplementation with 50 g/kg soybean oil significantly decreased the a* value of *Longissimus thoracis et lumborum*. Unexpectedly, supplementation with 2.5 g/h/day of probiotics notably increased the a* value of the same muscle. Moreover, 50 g/kg soybean oil also significantly reduced the value of a* and b* in perirenal fat. These results suggest that soybean oil supplementation promotes IMF deposition, which typically leads to a reduction in the a* value of muscle. Interestingly, when supplemented with 2.5 g/h/day of probiotics, the a* and chroma values increased, whereas the hue angle values showed a decrease, indicating a potential improvement effect of probiotics on color in goat meat.

In summary, we believe that supplementing soybean oil in the diet can increase the intake of fatty acids, which promotes the deposition of more fat in muscles and organs through microbial conversion, body absorption, and transport. Additionally, probiotic supplementation improves the composition of microorganisms in the body, particularly rumen microbes. Generally, this will make the fat deposition process more efficient, which will lead to lower a* values in the muscles. However, further investigation is required to understand why probiotics enhance the muscle a* value and to uncover the underlying mechanisms driving this effect.

### 3.4 Meat nutritional profiles

The incorporation of 50 g/kg soybean oil in goats’ diets could significantly increase (*p* = 0.05) the EE content in *B. brachii* and *Semimembranosus*. Interestingly, the WBSF values of the *B. brachii*, *Semimembranosus*, and *Longissimus thoracis et lumborum* were not affected (*p* > 0.05) by probiotics supplementation, while soybean oil supplementation reduced (*p* = 0.05) the WBSF values of the *Longissimus thoracis et lumborum* (Table 7). To our knowledge, the increase in DMI observed in this study likely led to higher oil intake, which may have enhanced the fat deposition process by increasing the activity of lipogenic enzymes and the fat synthesis rate in body tissues (Saqhir et al., 2012). However, the elevated EE content in the *B. brachii*, *Semimembranosus*, and *Longissimus thoracis et lumborum* supports this perspective. Notably, the increase in EE indirectly reflects the rise in IMF, which contributes to reduced WBSF values of the muscle and improves its tenderness. At this stage, the goat meat is also more tender. In summary, the tenderness of the meat is positively correlated with the IMF content and negatively correlated with the WBSF value (Pannier et al., 2018; Park et al., 2015; Picard et al., 2018). Consequently, the higher EE value observed in this study further explained the lower WBSF value of the *Longissimus thoracis et lumborum*.

**TABLE 7:** Effects of soybean oil and probiotics on chemical composition (g/100 g) and WBSF value of goat muscle.

Items	B_1_			B_2_				*p*‐value			
	P_1_	P_2_	P_3_	P_1_	P_2_	P_3_	SEM	B	P(L)	P(Q)	B×P
*Biceps brachii*											
DM	25.11	25.36	25.25	25.94	26.41	26.22	1.17	0.19	0.92	0.89	0.98
OM	96.04	96.15	95.94	96.12	96.24	96.23	0.26	0.25	0.71	0.79	0.87
EE	9.24**^B^**	10.02**^B^**	10.85**^B^**	11.36**^A^**	12.04**^A^**	12.75**^A^**	0.62	0.05	0.55	0.47	0.99
CP	70.71	79.71	79.41	73.94	73.45	77.46	4.35	0.52	0.10	0.32	0.35
WBSF (N)	65.15	66.74	58.31	61.54	53.31	63.01	3.21	0.73	0.26	0.67	0.40
*Semimembranosus*											
DM	25.54	26.15	26.06	26.14	26.64	26.07	0.87	0.48	0.63	0.75	0.91
OM	96.02	96.24	96.05	96.14	96.32	96.06	0.28	0.52	0.14	0.69	0.95
EE	9.61**^B^**	11.45	11.97**^B^**	11.58**^A^**	12.11	13.84**^A^**	1.40	0.05	0.05	0.26	0.59
CP	75.12	77.84	77.98	76.21	75.94	77.65	3.54	0.84	0.70	0.34	0.83
WBSF (N)	50.08	65.17	52.43	55.27	45.67	57.62	2.89	0.09	0.45	0.72	0.32
*Longissimus thoracis et lumborum*											
DM	25.91	26.92	26.73	26.24	26.90	25.81	1.18	0.76	0.59	0.66	0.77
OM	96.02	96.42	96.04	96.33	96.31	95.94	0.26	0.93	0.15	0.74	0.60
EE	9.41	11.62	11.64	12.51	10.52	11.53	1.31	0.07	0.61	0.28	0.81
CP	79.54	76.05	76.53	78.64	77.80	78.31	3.63	0.68	0.70	0.42	0.81
WBSF (N)	42.53	55.08**^A^**	48.31**^A^**	42.24	42.34**^B^**	46.75**^B^**	0.85	0.05	0.43	0.38	0.88

*Note*: Different superscript letters within the same row denote significant differences, where lowercase letters (a, b, c…) represent probiotic supplementation levels and uppercase letters (A, B…) represent soybean oil supplementation levels.

Abbreviations: CP, crude protein; DM, dry matter; EE, ether extract; OM, organic matter; SEM, standard error of the mean; WBSF, Warner–Bratzler shear force.

### 3.5 Fatty acid profiles

Fifty gram per kilogram soybean oil significantly increased (*p* = 0.02) the content of C18:3n3. Importantly, incorporating 50 g/kg soybean oil into goat diets resulted in a more pronounced increase (*p* < 0.01) in the content of C18:c9, t11, C18:t10, C12 CLA, and C20:2 fatty acids. Notably, the supplementation of the diet with probiotics significantly reduced the content of C22:0 fatty acids (*p* < 0.01). The TCLA content ranged from 0.48 to 0.81 g/100 g, with soybean oil supplementation significantly increasing TCLA content (*p* < 0.01). Moreover, the n‐6/n‐3 ratio ranged between 3.8 and 5.1, and 50 g/kg soybean oil supplementation significantly reduced (*p* < 0.01) this ratio (Table 8).

**TABLE 8:** Effects of soybean oil and probiotics supplementation on fatty acid profile in goat meat (g/100 g).

Items	B_1_			B_2_				*p*‐value			
	P_1_	P_2_	P_3_	P_1_	P_2_	P_3_	SEM	B	P(L)	P(Q)	B × P
C12:0	0.32	0.31	0.33	0.42	0.31	0.35	0.05	0.15	0.63	0.72	0.86
C14:0	4.84	4.62	4.83	5.01	4.82	4.82	0.50	0.37	0.88	0.38	0.67
C15:0	0.53	0.67	0.74	0.61	0.72	0.61	0.06	0.14	0.72	0.69	0.57
C16:0	21.91	19.70	20.00	19.94	20.65	19.24	1.40	0.43	0.44	0.26	0.39
C16:1	0.61	0.43	0.51	0.52	0.46	0.51	0.18	0.78	0.50	0.61	0.71
C17:0	4.12	4.84	5.48	4.54	4.81	4.72	0.80	0.86	0.51	0.75	0.67
C17:1	0.61	0.52	0.54	0.80	0.65	0.54	0.09	0.94	0.10	0.58	0.50
C18:0	11.91	12.88	13.77	11.51	13.01	13.52	1.40	0.90	0.39	0.66	0.97
C18:1n9	37.22	38.21	37.11	39.03	38.74	38.97	1.07	0.46	0.30	0.73	0.27
C18:2n6t	10.12	10.54	10.06	11.25	11.01	10.91	1.67	0.27	0.59	0.39	0.58
C20:0	0.30	0.10	0.21	0.21	0.20	0.21	0.03	0.39	0.10	0.78	0.29
C18:3n3	1.72**^B^**	1.61**^B^**	1.84**^B^**	2.21**^A^**	2.34**^A^**	2.22**^A^**	0.41	0.02	0.24	0.69	0.19
C18:c9,t11	0.41**^B^**	0.62	0.64**^B^**	0.72**^A^**	0.61	0.73**^A^**	0.02	<0.01	0.14	0.32	0.33
C18:t10,c12	0.05**^Bb^**	0.05**^Bb^**	0.07**^aBb^**	0.07**^Aab^**	0.09**^Aa^**	0.09**^Aa^**	0.01	<0.01	0.11	0.27	0.96
C20:2	1.01**^B^**	0.92**^B^**	0.85**^B^**	1.11**^A^**	1.12**^A^**	1.01**^A^**	0.07	<0.01	0.03	0.46	0.36
C22:0	0.07**^b^**	0.13**^a^**	0.11**^ab^**	0.09**^b^**	0.10**^ab^**	0.10**^ab^**	0.01	0.13	<0.01	0.51	0.10
C20:3n	0.62	0.61	0.63	0.71	0.64	0.52	0.05	0.89	0.10	0.68	0.34
TCLA	0.48**^B^**	0.64**^B^**	0.65**^B^**	0.74**^A^**	0.73**^A^**	0.81**^A^**	0.04	<0.01	0.10	0.60	0.37
∑ SFA	42.91	43.22	43.33	43.91	45.04	43.12	1.18	0.36	0.68	0.55	0.55
∑ PUFA	14.81	14.72	14.77	15.91	14.72	14.96	0.21	0.66	0.79	0.71	0.58
∑ DFA	64.51	66.24	64.81	66.12	66.33	65.64	2.5	0.94	0.97	0.76	0.25
n−6/n−3	5.01**^A^**	5.12**^A^**	4.88**^A^**	4.11**^B^**	3.82**^B^**	4.13**^B^**	0.20	<0.01	0.75	0.63	0.61
PUFA/ SFA	0.42	0.32	0.31	0.42	0.38	0.41	0.03	0.74	0.43	0.29	0.35

*Note*: Different superscript letters within the same row denote significant differences, where lowercase letters (a, b, c…) represent probiotic supplementation levels and uppercase letters (A, B…) represent soybean oil supplementation levels.

Abbreviations: DFA, desirable fatty acids (C18:0+ unsaturated fatty acids); PUFA, poly‐unsaturated fatty acids; SEM, standard error of the mean; SFA, saturated fatty acids; TCLA, total conjugated linoleic acids.

The results of this study suggest that probiotic supplementation may further enhance rumen biohydrogenation in the rumen, leading to increased CLA levels (primarily C18:c9,t11 and C18:t10,c12) in muscle (Kuhl & De Dea Lindner, 2016). Biohydrogenation is a key pathway for CLA biosynthesis by rumen microorganisms. This process involves the gradual hydrogenation of unsaturated fatty acids, such as linoleic and CLA, into saturated fatty acids by rumen microorganisms (Jenkins et al., 2008). Numerous studies have reported that supplementing diets with *S. cerevisiae* and *L. acidophilus* increases the abundance of fiber‐decomposing bacteria in the rumen, including *Ruminococcus albus*, *R. flavefaciens*, *F. succinogenes*, and *Butyrivibrio fibrisolvens* (Cherdthong et al., 2021; Lettat et al., 2012; Phesatcha et al., 2021; Zhu et al., 2017). These fiber‐decomposing bacteria are the main bacterial groups that undergo biohydrogenation in the rumen. Among them, *B. fibrisolvens* plays a critical role in this process, particularly in converting LA into c9‐t11 CLA (rumenic acid), which is subsequently transformed into C18:1 t11 (trans‐oleic acid) and eventually into C18:0 (stearic acid) (Jenkins et al., 2008; Paillard et al., 2007; Vasta et al., 2010). However, not all CLA is fully hydrogenated and converted to C18:0. Following CLA formation in the rumen, it is absorbed by enterocytes lining the small intestine and then enters general circulation for distribution. Some CLA is absorbed into the blood and deposited in the muscle tissue and mammary glands (Andrade et al., 2012; Badawy et al., 2023).

In this study, we speculate that soybean oil, as a rich source of LA, coupled with the addition of probiotics, increased the DMI of goats, allowing a substantial amount of fatty acid to enter the rumen. Under the influence of *S. cerevisiae* and *L. acidophilus*, the rumen microbial composition shifted, promoting the growth and activity of fiber‐decomposing bacteria and enhancing biohydrogenation efficiency. Consequently, a large amount of LA was converted into CLA, which was absorbed into the systemic circulation through the intestine, resulting in greater CLA deposition in muscle tissue.

### 3.6 Nutritional quality index

The incorporation of probiotics into goat diets could significantly reduce (*p* = 0.03) the TI index of goat meat. In contrast, both 50 g/kg soybean oil (*p* = 0.03) and probiotics (*p* = 0.04) significantly increased the NVI index of goat meat. There was no functional interaction between soybean oil and probiotics on all nutritional indices (*p* > 0.05) (Table 9). Nutritional indices in food, such as atherogenic index (AI) and TI, are critical as they account for the potential effects of food on human health. These indices are closely related to the risk of developing atherosclerosis and thrombosis (J. Chen & Liu, 2020; Pretorius & Schönfeldt, 2021). Lower AI and TI values mean that they contain more antiatherosclerotic and antithrombotic fatty acids, which may help prevent such diseases (Arruda et al., 2012). In this study, probiotics reduced the TI value. Although there are no established recommended TI ranges for goat meat, the results of this study show values lower than those of other meats, such as chicken (TI, 1.14) (Del Puerto et al., 2017); beef (TI, 1.86) (Mapiye et al., 2011); and pork (TI, 1.12) (Kasprzyk et al., 2015). Furthermore, as a key fatty acid quality indicator in animal‐derived foods, the NVI value reflects the overall quality of fatty acids in meat (Y. Chen et al., 2016). However, this study concluded that soybean oil and probiotics significantly increased the NVI value in goat meat. In conclusion, it was shown that soybean oil and probiotics could improve the overall quality of goat meat, and the produced goat meat may be more beneficial to human health.

**TABLE 9:** Effects of soybean oil and probiotics supplementation on nutritional quality index in goats.

Items	B_1_			B_2_			SEM	*p*‐value			
	P_1_	P_2_	P_3_	P_1_	P_2_	P_3_		B	P(L)	P(Q)	B × P
AI	0.75	0.69	0.72	0.68	0.70	0.67	0.04	0.17	0.17	0.09	0.24
TI	1.19**^a^**	1.13**^ab^**	1.17**^a^**	1.00**^b^**	1.07**^ab^**	1.06**^b^**	0.02	0.64	0.03	0.15	0.71
NVI	2.24**^B^**	2.59	2.54**^B^**	2.53**^A^**	2.51	2.73**^A^**	0.04	0.03	0.04	0.25	0.38
HFI	2.05	2.05	2.05	2.18	2.14	2.17	0.02	0.72	0.69	0.51	0.64
HH	1.92	2.15	2.06	2.17	2.07	2.20	0.08	0.64	0.28	0.48	0.41

*Note*: Different superscript letters within the same row denote significant differences, where lowercase letters (a, b, c…) represent probiotic supplementation levels and uppercase letters (A, B…) represent soybean oil supplementation levels.

Abbreviations: AI, atherogenic index; HH, hypocholesterolemic/hypercholesterolemic; HFI, healthy fatty Index; NVI, nutritive value index; SEM, standard error of the mean; TI, thrombogenic index.

## 4 CONCLUSIONS

In this study, we investigated the effects of dietary supplementation with soybean oil and probiotics on meat quality and CLA content in the muscles of crossbred goats. Under the current experimental conditions, supplementation with 50 g/kg soybean oil and 2.5 g/h/day probiotics has the best effect on improving goat meat quality and fatty acid composition, especially increasing the CLA content in goat meat. Additionally, these supplements also significantly enhanced DMI, ADG, and carcass yield in goats. Consequently, soybean oil and probiotics are promising methods for enhancing goat growth performance and improving meat quality. Nevertheless, further research is needed to determine the molecular mechanisms by which the supplementation of soybean oil and probiotics affects meat quality and fatty acid distribution. Notably, the study of the mechanism of whether there is a synergistic effect or interaction between soybean oil and probiotics is also one of the issues that still need to be addressed in the future.

## AUTHOR CONTRIBUTIONS

**Yong Han**: Conceptualization; methodology; resources; writing—original draft; writing—review and editing; project administration; supervision. **Defeng Wang**: Conceptualization; investigation; validation; supervision; writing—review and editing. **Wen Xiao**: Data curation; writing—review and editing. **Chao Yuan**: Conceptualization; writing—review and editing; investigation. **Yang Yang**: Data curation; software; writing—review and editing. **Yong Long**: Writing—original draft; writing—review and editing; data curation; methodology.

## CONFLICT OF INTEREST STATEMENT

The authors declare no conflicts of interest.

## ETHICS STATEMENT

All animal procedures were conducted meticulously in strict adherence to animal welfare guidelines, under the rigorous oversight of the Experimental Animal Ethics Committee of Guizhou University in Guizhou, China (EAE‐GZU‐2020‐E025). The study was conducted in strict adherence to local legislation and institutional regulations.

## Data Availability

Readers may request the corresponding author to provide data supporting the findings of this study, under reasonable circumstances.
